# Killer whales redistribute white shark foraging pressure on seals

**DOI:** 10.1038/s41598-019-39356-2

**Published:** 2019-04-16

**Authors:** Salvador J. Jorgensen, Scot Anderson, Francesco Ferretti, James R. Tietz, Taylor Chapple, Paul Kanive, Russell W. Bradley, Jerry H. Moxley, Barbara A. Block

**Affiliations:** 1 Monterey Bay Aquarium, 886 Cannery Row, Monterey, CA 93940 USA; 2 Department of Biology, Stanford University Pacific Grove, California, 93950 USA; 3 Point Blue Conservation Science, 3820 Cypress Drive #11, Petaluma, CA 94954 USA; 4 Fish and Wildlife Management, Montana State University, PO Box 173460, Bozeman, MT 59717 USA

## Abstract

Predatory behavior and top-down effects in marine ecosystems are well-described, however, intraguild interactions among co-occurring marine top predators remain less understood, but can have far reaching ecological implications. Killer whales and white sharks are prominent upper trophic level predators with highly-overlapping niches, yet their ecological interactions and subsequent effects have remained obscure. Using long-term electronic tagging and survey data we reveal rare and cryptic interactions between these predators at a shared foraging site, Southeast Farallon Island (SEFI). In multiple instances, brief visits from killer whales displaced white sharks from SEFI, disrupting shark feeding behavior for extended periods at this aggregation site. As a result, annual predations of pinnipeds by white sharks at SEFI were negatively correlated with close encounters with killer whales. Tagged white sharks relocated to other aggregation sites, creating detectable increases in white shark density at Ano Nuevo Island. This work highlights the importance of risk effects and intraguild relationships among top ocean predators and the value of long-term data sets revealing these consequential, albeit infrequent, ecological interactions.

## Introduction

High trophic-level consumers, or top predators, play an important ecological role through top-down forcing^1–3^. Regulation of prey density via direct consumption by predators is the most common form of top-down control documented in ecological literature^4–6^. Yet there is also increasing recognition for non-lethal or behaviorally mediated mechanisms, which can similarly shape ecosystem function and structure^7–11^. Non-consumptive mechanisms include ‘risk effects’ in which prey are not removed, but respond behaviorally to the presence of a predator by reducing activity or shifting habitats to reduce risk^9^. Risk effects can result in the ecological equivalent of density reduction and may have negative impacts on fitness including decreased reproductive success through loss of foraging opportunities, increased stress, and increased energy demands associated with predator avoidance^12–14^. Top-down forcing can result in trophic cascades where changes in predator forcing alter the densities of intermediate and lower level consumers down through multiple trophic levels^15^. Risk effects can also initiate trophic cascades^8,16,17^. For instance, under threat from a potential predator, behavioral responses of a risk-averse intermediate consumer can result in the local release of its food base^18^.

In marine ecosystems predator-prey interactions and resulting ecological effects have received relatively more focus^2,6,19^, whereas much less is known about the interactions among top consumers^20^, which likely have similar top-down implications for marine ecosystems^21^. Large-bodied upper trophic-level consumers have few natural predators. However, competition within predator guilds can lead to complex interactions and strongly affect the distribution and abundance of the predator populations^5,21–23^. Relatively common in terrestrial systems, intraguild predation among top predators can potentially reduce exploitation competition for food resources and confer energetic benefits for the prevailing consumer^23,24^. Even in cases where the rate of killing is very low^8^, indirect dominance effects can profoundly influence the behavior and fitness of sub-dominant predators^25–27^. In spite of their ubiquity where well-studied^22,23,28^, the frequency and importance of lethal and sub-lethal interactions among top marine predators remain difficult to measure in the oceanic realm and therefore are potentially underrepresented^3^.

Many marine top predators exhibit migratory behavior and seasonal aggregations at foraging areas^29^. Concentrated seasonal foraging is crucial in supporting migratory behavior in many consumers^30,31^ and, conversely, the seasonal influx of predators can have strong regulatory and behavioral effects on local prey populations^3^. Perturbations in such predator-prey systems may therefore be impactful for both prey and predator with potentially cascading effects^1^. In cases of co-occurrence between top predators at such sites, the effect of intraguild interactions on local ecosystem dynamics remains relatively unknown.

Here we document and investigate interactions between two top ocean predators, white sharks (*Carcharodon carcharias*) and killer whales (*Orcinus orca*). In the northeastern Pacific (NEP) white sharks aggregate seasonally at Southeast Farallon Islands (SEFI), Año Nuevo Island (ANI), and other pinniped rookeries off the west coast of North America^32,33^. The sharks’ timing and observed foraging is associated with the seasonal haulout of juvenile elephant seals (*Mirounga angustirostrous*), a preferred prey^34–37^ consumed prior to offshore migration^32,38^. Although white sharks also forage or scavenge on cetaceans^39^, teleosts, other elasmobranchs^40^ and various pinnipeds, their seasonal targeting of elephant seals^41^ provides a consistent source of caloric capital to fuel extended oceanic migrations^42,43^.

**Figure 1:**
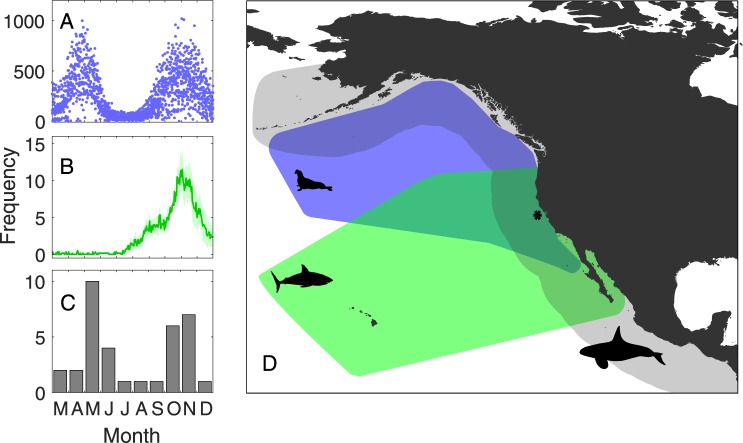
Spatial and temporal overlap of two top predators, white sharks (*Carcharodon carcharias*), and killer whales (*Orcinus orca*), and their shared prey, juvenile elephant seals (*Mirounga angustirostrous*), in the Northeastern Pacific (see Supplement) and at Southeast Farallon Islands (SEFI). Seasonally concentrated activity of each species at SEFI (*) evident from (**A**) weekly *M*. *angustirostrous* counts between March and December (1987–2013), (**B**) daily mean number of tagged *C*. *carcharias* detected (2007–2013) with shaded standard error and, (**C**) monthly frequency of *O*. *orca* observed (1987–2013). Note the two predators co-occur only during the fall peak. Map was created using R software (v3.5.1; https://www.R-project.org/).

White sharks and killer whales exhibit a high degree of niche overlap along the western shores of North America. The coastal distribution of NEP white sharks extends from northern Mexico to Canada (and in El Nino years up into Alaskan waters), entirely within the coastal distribution of NEP killer whales that range from Mexico to the Aleutian islands of Alaska^44^ (Fig. 1). NEP killer whale populations form distinct and stable social groups (pods), which differ in specialization of prey choice (ecotypes). Three recognized NEP ecotypes, ‘resident,’ ‘transient,’ and ‘offshore’, exhibit genetic and phenotypic differentiation^45–48^. Transient pods typically feed on marine mammals including elephant seals and sea lions, whereas resident pods target teleosts, mainly salmonids^45,49^. The offshore ecotype is least known, but thought to primarily target teleosts including salmonids and elasmobranchs such as Pacific sleeper sharks^45,50^. All three killer whale ecotypes overlap spatially with NEP white sharks and share similar prey resources, and thus may be considered part of the same ecological guild^51^. Regional overlap is highest during fall and early winter, when NEP white sharks show high site fidelity and extended residency periods at SEFI or other coastal aggregation sites near pinniped rookeries^32^ for approximately 4–4.5 months (Fig. 1).

Despite an extensive overlap in distributional range and trophic niche, observations of direct interactions between killer whales and white sharks are extremely rare, but have been recorded off California, South Africa, and Southern Australia^52,53^. A clear understanding of the ecological relationship between these two top predators has remained elusive. An interaction between these top predators in the NEP was documented on Oct 4, 1997, at SEFI in which a white shark was killed and partially consumed (liver only) by transient killer whales. Immediately following this event, observations of white sharks during regular surveys at SEFI declined precipitously; only two predations by sharks were observed in the remaining eight weeks of study at SEFI^52^.

In the current study we use a combination of an extensive electronic tagging dataset of white sharks throughout Central California together with long-term observational surveys of shark-pinniped predatory interactions and killer whale occurrences at SEFI to elucidate the frequency and consequences of rarely observed intraguild interactions between white sharks and killer whales. We reveal in detail the immediate behavioral nature of the predator interactions, as well as resulting effects that white shark redistribution has on the predator-prey relationship between white sharks and elephant seals.

## Materials and Methods

### White shark tagging

Between 2006 and 2013, we tagged 165 white sharks (*Carcharodon carcharias*) with acoustic tags (V16-4H-A69, Vemco; transmitting at 158 Db every 60–180 s for >1400 days) in the vicinities of Southeast Farallon Island, Tomales Point, and Año Nuevo Island using previously described methods^32^. Briefly, white sharks were attracted to the research boat using a seal decoy made from outdoor carpet. Upon investigation by a shark, the decoy was retrieved using a fishing rod, reel and monofilament line. A small amount of olfactory attractant served to retain the shark near the boat, while the tag was applied using 3 to 4 m pole to insert a titanium dart tethered to the tag beneath the sharks’ dorsal skin. All methods were carried out in accordance with relevant guidelines and regulations, and all experimental protocols were approved under Stanford University animal care protocol 10765. The annual probability of tag loss (shedding) was estimated at 0.32 (95% CI = 0.26, 0.39)^54^, and annual incremental tagging (average 20 per year) provided a relatively constant flux of tagged individuals in the study system. We maintained continuous coverage in tag detection throughout the study period using sub-surface moored acoustic receivers (VR3, Vemco) stationed at the same three tagging locations^32^. Equivalent acoustic receivers (VR3, Vemco) deployed off Point Reyes by other research teams provided opportunistic detections supplementing the data collected at the primary shark aggregation sites.

### Long-term surveys at Southeast Farallon Island

We recorded the number of hauled-out elephant seals from weekly elephant seal population census surveys conducted throughout the duration of the study (1987–2013) under NMFS Permit No. 373-1868. Surveys occurred between 1000 and 1600, throughout the year. The number of individuals hauled-out on SEFI were tallied with respect to age class as previously described in depth^55^. During the same period, we recorded the number of predations by white sharks from annual ‘shark watch’ surveys conducted from a lighthouse platform at the highest peak of SEFI (elevation 90 m). Between September 1 and November 30, trained observers continually scanned the waters around the islands for the occurrence of predation events during all daylight hours, unless visibility dropped below 1.6 km, winds exceeded 25 knots, or rain was persistent^37^. Observation hours averaged 569.7 hours per year (SD = 93.6). Predation observations included prey species when identifiable. Observational survey data were non-experimental, and carried out in accordance with relevant guidelines and regulations.

Killer whale observations were aggregated from a variety of sources, including the ‘shark watch’ surveys, island cetacean surveys^56^, opportunistic island-based observations, and reports received via a network of wildlife-viewing tour boats during daily radio contact. Killer whale observations were not procedurally standardized, however were relatively consistent throughout the year, except between September 1 and November 30 each year when observation effort increased during the standardized ‘shark watch’ survey. When possible, an estimate of the minimum distance between killer whale pods from the nearest point of the island was recorded. If observation notes indicated killer whales were inside one of the island bays, then a minimum distance of <1 km was assigned. For survey observations with no killer whale sightings, we assumed a distance value of >15 km, a distance beyond the maximum range included in SEFI cetacean surveys^56^. Where distance was missing from killer whale sightings, we assumed an average distance obtained from all other distance values. Accounts of killer whale behavior and photographs of individual killer whales including dorsal fin and saddle pigmentation were collected from tour boat operators and Island staff. Photos were compared to killer whale ID catalogues to match individuals and determine the pod’s ecotype and size.

### Analysis

We hypothesized that shark predations on elephant seals would decline in years when killer whales occurred at SEFI. We initially fit a log-log regression model to the positive predator-prey relationship^37^ between annual predation rate (number of predations per observation hour) by sharks and mean fall (September 1–November 30) elephant seal counts to determine if deviations corresponded with killer whale occurrences. To test whether the presence of killer whales disrupted the seasonality of shark predations on elephant seals, we modeled daily predation events using a Generalized Additive Mixed Model. We assumed that the realized number of predation events followed a Poisson distribution and that the expected number of predations per day was a smooth function (cyclic cubic regression spline) of date included as ordinal days within a season from September 1 to November 30. We also constrained the spline to start and end at similar values. We treated sighting distance as a factor variable and as a quadratic function of survey observation effort in hours^37^. We also hypothesized that the shape of the functional relationship between daily shark predations on seals and date would change in relation to the distance of sighted killer whales, and consequently affect the average seasonal predation rate by white sharks. Therefore we included an interaction term between the spline of date and distance. We also expected to have between-year variability in predation rate. This could arise from annual variability in the number or behavior of individual sharks and seals. To control for this variability and ensure unbiased parameter estimates on the other terms, we included season (categorical) as a random effect, modeled as a smooth term with a random effect spline basis^57^. For model fitting we used a restricted maximum likelihood approach with the package “mgcv” in R^57^.

To test the hypothesis that killer whale activity in close proximity to SEFI elicited avoidance behavior, we looked at the number of tagged sharks detected per day at each site (SEFI, ANI, and TOM) between 2006 and 2013. We compared this metric during periods when killer whales were observed at SEFI against the mean value for all other years.

## Results and Discussion

### Multi-predator community at Southeast Farallon Island (SEFI)

Long-term intensive monitoring surveys, combined with electronic tagging and observational studies, revealed the frequency and modality of cryptic interactions amongst marine predator populations at SEFI. Seasonality in white shark and killer whale presence matched annual cycles in prey aggregations, namely juvenile (age 0–3) northern elephant seals (*Mirounga angustirostris*) that first haulout during spring molt (peaking in April and May) and then again in the fall (peaking in October and November) (Fig. 1). An estimated 219 adult and sub-adult white sharks ((130, 275) 95% credible intervals) aggregate and feed at SEFI and adjacent elephant seal rookeries around Point Reyes during this fall haulout period^58^. Long-term (1972–2010) birth rates of elephant seals at SEFI are variable (median = 198 births/year; mean = 232; SD = 132) and have decreased to a relatively stable level over the past decade, while the regional population continues to rapidly increase^59^. Additionaly, California sea lions (*Zalophus californianus*), Steller sea lions (*Eumetopias jubatus*), harbor seals (*Phoca vitulina ricardii*), and northern fur seals (*Callorhinus ursinus*) also haul out at SEFI at various times of the year^55^.

Killer whale pod observations occurred year-round on 57 occasions between 1987 and 2013 (Fig. 1C). These sparse occurrences peaked in May, concurrent with gray whale calf migrations, followed by a secondary fall peak during October and November (Fig. 1C). The co-occurrence of white sharks and killer whales was confined to fall, coincident with the peak in adult white shark activity at SEFI (Fig. 1B). During the fall overlap (September–November) killer whale pods were recorded during daily surveys ( mean = 7.7 hr/day; weather permitting) from the island lighthouse at various distances from SEFI on 18 out of 1998 survey days in eight different years: 1992, 1995–1998, 2000, 2001, 2009, and 2013 (Table 1). The recorded duration of these 18 visits ranged from a maximum of 5.5 hours to less than an hour. When killer whale ecotype could reliably be identified (n = 5), mammal-eating transient pods were the most common visitors to SEFI (four of five), while offshore individuals were identified on a single occasion in 2009 when both offshores and transients were observed (Table 1).

**Table 1:** Summary of killer whale observations during fall (September 1 – November 30) standardized surveys at Southeast Farallon Island between 1987 and 2013.

	Date	Minimum dist. (km)	Duration (hr)	Pod size	Predation observed	Shark flight	Ecotype
	22-Oct-92	—	—	6	no	no	—
	24-Sep-95	4.8	<5	12	no	no	—
	23-Nov-96	5.6	—	15	no	no	—
**	4-Oct-97	0.2	2.4	2	yes	Srv	Tran
	14-Oct-97	0.2	5.5	3	no	Srv	—
	17-Oct-97	—	—	2	no	Srv	Tran
	31-Oct-97	—	<1	2	no	Srv	Tran
	14-Oct-98	2.8	—	4	no	no	—
	14-Nov-99	7.4	—	17	no	no	—
	18-Nov-00	—	—	15	no	no	—
**	19-Nov-00	0.2	<4	12	yes	SW	—
	21-Nov-00	0.2	—	5	no	SW	—
	9-Nov-01	6.5	—	12	no	no	—
**	2-Nov-09	0.2	2.5	7	yes	Srv, Tag	Tran, Off
**	20-Nov-11	*	*	*	*	Srv, Tag	*
	4-Sep-12	2.8	—	1	no	no	—
**	31-Oct-13	0.6	—	13	no	Srv, Tag	—
	9-Nov-13	—	—	2	no	Srv, Tag	—
	11-Nov-13	—	—	2	no	Srv, Tag	—

A flight response by white sharks was documented from tag data (Tag) or standardized surveys (Srv) on four occasions when transient (Tran) or offshore (Off) killer whales occurred in close proximity to SEFI and on a fifth occasion when killer whales were not observed (surveys were also not conducted during inclement weather).

SW - evidence from shark watch data.

Tag - evidence from tagging data.

**Initial flight response by white sharks.

*Killer whales inferred but not observed (see SI figure for 2011).

**Figure 2:**
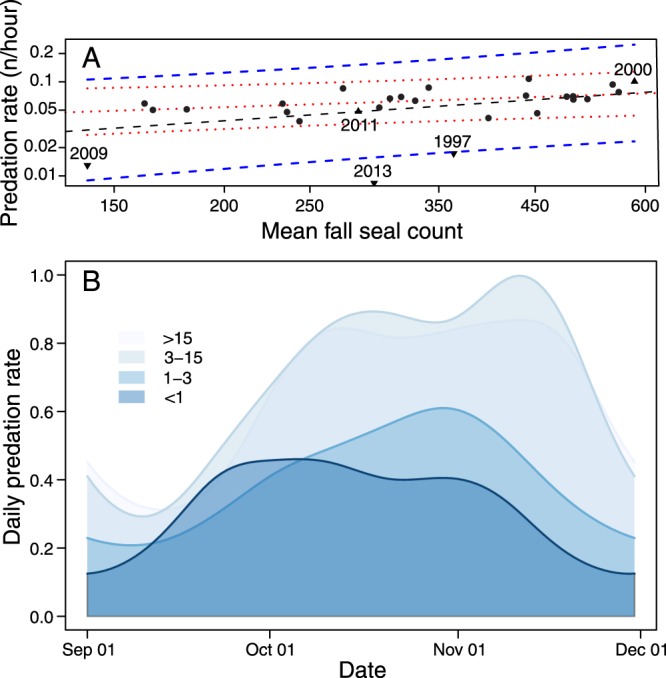
Predator-prey relationship between white sharks (*Carcharodon carcharias*) and elephant seals (*Mirounga angustirostrous*) altered by the presence of killer whales (*Orcinus orca*) at Southeast Farallon Island (SEFI). (**A**) Annual predation rate by *C*. *carcharias* as a function of mean fall (Sept. – Nov.) *M*. *angustirostrous* counts fit with a log-log regression line (dashed black line) showing confidence interval (dashed blue lines). Points are years where no flight response was detected, and triangles are the years in which a flight response was observed, near or before the peak of the *C*. *carcharias* season (≤November 2, inverted triangles), and near the end of the season (≥November 19, upright triangles). For comparison, an equivalent regression fit excluding flight years is shown with red dotted lines. (**B**) Seasonal *C*. *carcharias* kill rate as a function of the observed distance of *O*. *orca* activity to SEFI. The distribution of observed predations was reduced and truncated as a function of *O*. *orca* proximity to the common foraging ground (distance given in legend in km).

White sharks aggregated at SEFI annually, where the observed number of predations by sharks peaked during October and November (Fig. 2B). During the fall surveys, a mean of 40 observed predations (±16 SD; N = 27 years) by sharks occurred annually on elephant seals and unidentified pinniped prey. In years when killer whales were not observed or were sighted 3 or more km from shore (N = 19), the distribution of predation events on pinnipeds peaked between mid-October and mid-November (Fig. 2B). In years when killer whales were sighted <3 km from shore (N = 9), this predation rate was depressed and truncated (Fig. S3 and Table S1). Therefore, annual predation rates on pinnipeds were significantly impacted when killer whale activity occurred at a distance threshold <3 km from the SEFI seal haul-out.

Overall, the observed annual rate of predation by sharks was positively correlated with the abundance of elephant seals present (R^2^ = 0.191, p = 0.023) (Fig. 2A). However, in years when killer whales occurred in close proximity to the island during or before peak shark abundance, the observed rate of predation by sharks deviated most from this relationship dropping 3.5 to 7-fold from the long-term average of 6.02 ± 2.4 predations per 100hrs (SD), to 1.73, 1.29, and 0.84 respectively in 1997, 2009, and 2013 (Fig. 2B). In 2000 by contrast, killer whales also occurred close to SEFI, but much later in the season (November 18; Table 1) resulting in no deviation from the expected annual predation rate (Fig. 2A).

### Displacement of white sharks and flight response

**Figure 3:**
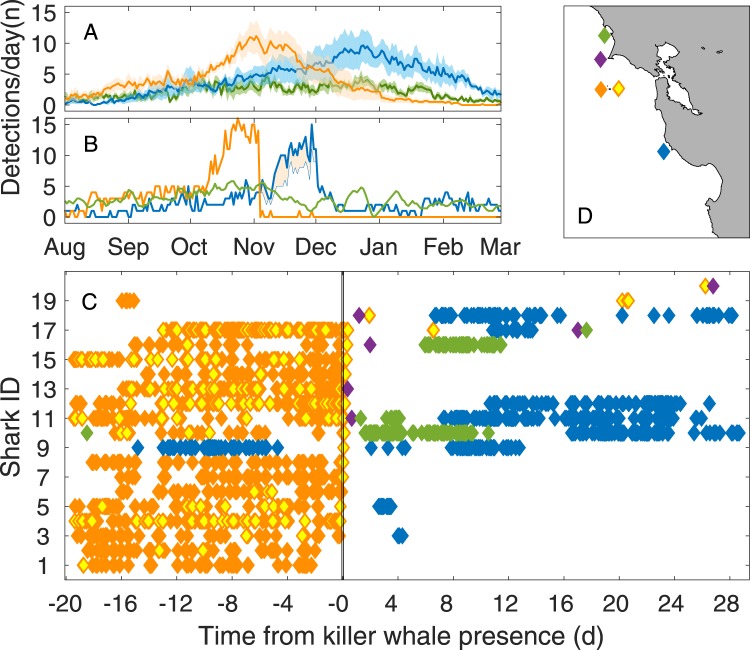
The flight response of white sharks (*Carcharodon carcharias*) triggered by the presence of killer whales (*Orcinus orca*) at a common foraging site, Southeast Farallon Islands (SEFI). (**A**) Mean daily number of acoustic tagged *C*. *carcharias* detected (2007–2013; excluding 2009; shaded standard error) at Central California receivers colored by location: Tomales Point (green), Southeast Farallon Islands (orange and orange/yellow), Año Nuevo Island (blue), and Point Reyes (purple). (**B**) The number of tagged *C*. *carcharias* detected per day at each site (respectively colored) during the 2009 season showing the sudden departure of all tagged individuals from SEFI in response to *O*. *orca* (Nov 2) presence. Note the subsequent influx around Año Nuevo Island where the shaded orange area represents individuals present at SEFI during killer whale interactions. (**C**) Detections of each tagged shark at color-coded locations are shown along the horizontal timeline illustrating the abrupt departure from SEFI by tagged *C*. *carcharias* following *O*. *orca* presence (between vertical black lines) and subsequent avoidance. Solid orange diamonds indicate the western SEFI receiver while orange with yellow centers indicate the eastern receiver. (**D**) Precise receiver locations are indicated by the right corner of each solid diamond and the left corner of the yellow filled diamond.

Acoustic tag detections documented the abrupt and consistent flight of white sharks from SEFI in 2009, 2011, and 2013 (Figs 3 and SI). In the best-documented instance, killer whales from two separate pods (offshore and transient ecotypes; Table 1) arrived at SEFI on November 2, 2009, when 17 previously tagged white sharks were present. Killer whales were present at SEFI for just over 2.5 hours between 12:48 and 15:30 local time, remained on the western side of SEFI during approach and initiated three separate killing bouts on pinnipeds, then departed to the north. There were no observations of direct predation on white sharks, and all tagged animals were later confirmed alive through acoustic detections; still predations on untagged white sharks could not be ruled out.

Desertion of SEFI by all tagged sharks followed the foraging behavior of killer whales close to SEFI. Regular daily detections of 17 tagged animals at two stationary acoustic receivers moored on eastern and western sides of SEFI (SI) discontinued abruptly following the appearance of killer whales (Fig. 3). Overall, the mean number of white sharks detected per day at SEFI declined from a seasonal maximum to zero for the remainder of the season. Declines in detections followed a spatial gradient, immediately subsiding at the western receiver most proximal to killer whale observations, followed by a tapering of detections over the following hours at the eastern receiver (Fig. 3). Seven hours and 50 minutes following the event, no tagged sharks remained within receiver range at SEFI and 16 individuals (of 17 displaced tagged sharks) were not detected at SEFI again until the following season (July 2010 or later). One individual returned a week later (November 8), and was detected at SEFI three times over 73 minutes, before departing and being re-detected at Año Nuevo Island (ANI) on November 24.

Anomalous shark absences at SEFI for the remainder of the 2009 season resulted in influxes of displaced individuals at mainland aggregation sites. Within 2 to 13 days of departing SEFI following the killer whale disturbance at SEFI, seven tagged individuals relocated nearly 90 km to the south at ANI. Three individuals were redetected at Tomales Point for extended periods, before two of these continued to ANI (Fig. 3). These influxes at mainland sites resulted in daily totals of individual sharks detected at ANI increasing sharply from 4 day^−1^ on 2 November to 10 day^−1^ by 14 November and peaking at 16 day^−1^ by 23 November. In contrast, SEFI remained virtually shark-free for the remaining season. Three tagged individuals not initially present during the killer whale event were detected subsequently at SEFI, though for abbreviated durations (0.25, 1, and 11 hours, respectively) compared to mean SEFI residency periods of 35 days^32^. Two of these three sharks were then subsequently detected at mainland aggregation sites (Fig. 3).

Acoustic tag records provided a clear ‘signature’ for understanding and estimating flight responses of sharks relative to other killer whale occurrences at SEFI during years when sufficient active tags were present (see Table 1). The well-documented incident in 2009 was consistent with previous observations of cessation of seal predation at SEFI by white sharks following brief killer whale visits^52,60^. Acoustic tag records revealed two additional similar signatures at SEFI: November 20, 2011, with 10 tagged individual present, and October 31, 2013, with 3 tagged individuals present (see SI). Inclement weather resulting in poor visibility precluded visual confirmation of killer whales for the former event (no surveys that day) in 2011. On the 2013 occasion, 13 killer whales were observed during regular shark visual surveys from SEFI. In both cases following typical flight responses, no further tag detections were recorded at SEFI for the remainder of the season (see SI). Similarly, no further predations of pinnipeds by white sharks were observed during the remaining visual surveys in 2011 and 2013, and only a single predation during the remaining season in 2009 near Mid-Farallon Island, 3.4 km northwest of SEFI. In summary, a white shark flight response from SEFI related to killer whale occurrence was identified in four separate years, along with a fifth flight response with unconfirmed attribution (Table 1). While intensive observer survey data are lacking at TOM and ANI, no equivalent flight response was ever apparent in acoustic tagging data with continuous coverage between 2006 and 2013.

### Ecological roles and context of killer whale-shark interactions

Transient killer whales were present in the two flight response years when ecotypes could be reliably determined (1997, 2009), whereas offshore individuals were identified in addition to transients in the 2009 disturbance. In determining the ecological relationship between white sharks and killer whales, understanding whether their interactions are defined by predator-prey or competitive aggression interactions depends on killer whale ecotype. Mammal-eating transient killer whales^45^ are direct competitors, but also pose a predatory threat as illustrated in the 1997 event^52^ (see introduction). Interactions with the offshore ecotype are potentially predatory, as well as competitive. Offshore killer whales are known to forage on teleosts and elasmobranchs, the latter forming a potentially important dietary component as evidenced by apical teeth worn flat, presumably from the abrasive shark skin^50^, and observations of repeated feeding on Pacific sleeper sharks, *Somniosus pacificus*^61^. Residents are likely a weak competitor (for teleosts) and potentially not a predation threat^45,49^. Whether white sharks might distinguish a predatory versus competitive threat remains unknown, but the result may be the same. Like predation pressure, interspecific competitive aggression can similarly drive behavior that reduces encounter rates, shape habitat use, and shift activity schedules^8,62^. Only one direct predation on white sharks by killer whales was ever confirmed on white sharks at SEFI^52^, yet white sharks vacating SEFI, effectively freed up potential pinniped resources for the killer whales and restricted white shark access to those resources.

### Risk effects among top ocean predators

This study demonstrates the occurrence of risk effects among upper trophic level marine predators. The key interactions surrounding the phenomena remained cryptic and rarely observed despite intensive long-term visual surveys and multi-year continuous electronic tracking coverage. In the rare instances when both predators co-occurred at SEFI, antagonistic interactions between them resulted in the extended displacement of foraging white sharks via risk effects (Fig. 3), and in turn reduced local predation pressure on seals (Fig. 2). Despite exceptionally brief killer whale visits to SEFI (2.4–5 hr) during well-documented events near the peak white shark foraging season, the observed predation rate on pinnipeds by white sharks during those years decreased (Fig. 2). It is unlikely that killer whale predation on pinnipeds could compensate for the reduction in predation by white sharks following their displacement. Of the predation events observed at the surface in 15,383 hours of lighthouse surveys between 1987 and 2013, 912 were attributed to white sharks and only 5 events on 3 dates to killer whales.

Killer whales exert top-down effects in various systems by directly reducing meso-predator density through consumption^1,4^ as well as eliciting shifts in prey behaviors and distributions due to risk effects^63,64^. Similarly, large sharks can have a direct regulatory influence over their prey populations^6,65,66^, and induce food-safety tradeoffs^67^ including avoidance behavior^68,69^. This study suggests that intraguild interactions between killer whales and white sharks may result in cascading effects at lower trophic levels by reducing consumptive (and possibly non-consumptive) effects on elephant seals. Quantifying the indirect population-level effects killer whales induce on white sharks may have on elephant seals locally and regionally remains an important future direction. Northern elephant seals are undergoing rapid habitat expansion and population growth, following long-term human exploitation and extreme depletion^59^. Any population regulatory effects white sharks, killer whales, and their interactions have on elephant seals could become more significant as elephant seals approach an equilibrium level.

Occasional consumption of the highly-caloric liver of white sharks may confer ancillary energetic benefits to the killer whale. The fitness loss to white sharks from direct lethal interactions with killer whales is unambiguous. But avoidance behaviors in response to killer whale presence could also impact white shark fitness by restricting spatiotemporal access and activity to habitats that are sub-optimal or more competitive (more densely populated by conspecifics). Intimidation and predation risk pervasively affects entire populations, not just the individuals directly killed^16^. Potential consequences of displacement to white sharks should be evaluated within the ecological context of their migratory phenology. Fall-time aggregations and site fidelity of NEP white sharks along the central California coast immediately precede extensive offshore migrations to relatively oligotrophic waters^32,60,70^. Despite spending one third of their time in coastal California habitats, adults assimilate nearly half their protein from coastal foraging^41^. Concentrated energy acquisition during this coastal phase is stored in the oil-rich liver mass and is expended during long migrations (1000–3000 km) to seasonal offshore subtropical habitats^43^, where males increase diving activity extensively^32,38^. Disruptions of foraging prior to migration is known to negatively impact migratory performance in numerous long-distance migrating species^30,71,72^. Future efforts should aim to measure the impact and ecological implications of these risk effects on white shark fitness and elephant seal population dynamics.

## Supplementary information

Supplementary Information

## Data Availability

Data underlying this study and described above in Methods are archived (open access) at https://osf.io/b7su4/?view_only=f4f874c19e044ea5951a9ac355954d9f. These include (1) aggregated phenological acoustic tag detection data (2007–2013), and raw tag detection data (October 14 to November 30, 2009) (2) weekly census data of juvenile elephant seals at SEFI (1987–2013), and (3) killer whale observations at SEFI aggregated by month (1987–2013).
