# Supplementary material for: Killer whales redistribute white shark foraging pressure on seals
Source: Sci Rep. 2019 Apr 16;9:6153. doi: 10.1038/s41598-019-39356-2 (PMC6467992; doi:10.1038/s41598-019-39356-2)

SUPPLEMENTARY INFORMATION

Killer whales redistribute white shark foraging pressure on seals

Salvador J. Jorgensen*[1], Scot Anderson[1], Francesco Ferretti[2], Jim Tietz[3], Taylor Chapple[2], Paul Kanive[1,4], Russell W. Bradley[3], Jerry Moxley[1], Barbara A. Block[2]

[1]Monterey Bay Aquarium, 886 Cannery Row, Monterey, CA 93940, USA

[2]Department of Biology, Stanford University Pacific Grove, California 93950, USA

[3]Point Blue Conservation Science, 3820 Cypress Drive #11, Petaluma, CA, 94954, USA

[4]Fish and Wildlife Management, Montana State University, PO Box 173460, Bozeman, MT 59717, USA

Data Sources for Northeastern Pacific Species Range Maps

Shark and seal range maps were approximated determined from published movement data presented on white sharks (Weng et al. 2007, Domeier and Nasby-Lucas 2008, Jorgensen et al. 2010) and elephant seals (Robinson et al. 2012) respectively. Since these datasets are primarily Californian in focus, distributional extents were broadened to include known aggregations at and movements from Guadalupe Island, another primary co-occurrence site for these two species. Separately, range maps for killer whales were inferred from maps produced in the 2017 stock assessment report for the West Coast Transient stock (https://www.fisheries.noaa.gov/webdam/download/82960328), as well as Figure 4 in Wiles et al. 2004 that encompasses both transient and offshore ecotypes (https://www.researchgate.net/profile/Gary_Wiles/publication/242367775_Washington_State_status_report_for_the_killer_whale/links/00b49539e5d1ed4ea3000000.pdf). Importantly, the pelagic boundary of the ranges is ill-defined, due to declining observations out to sea.

Figure S1

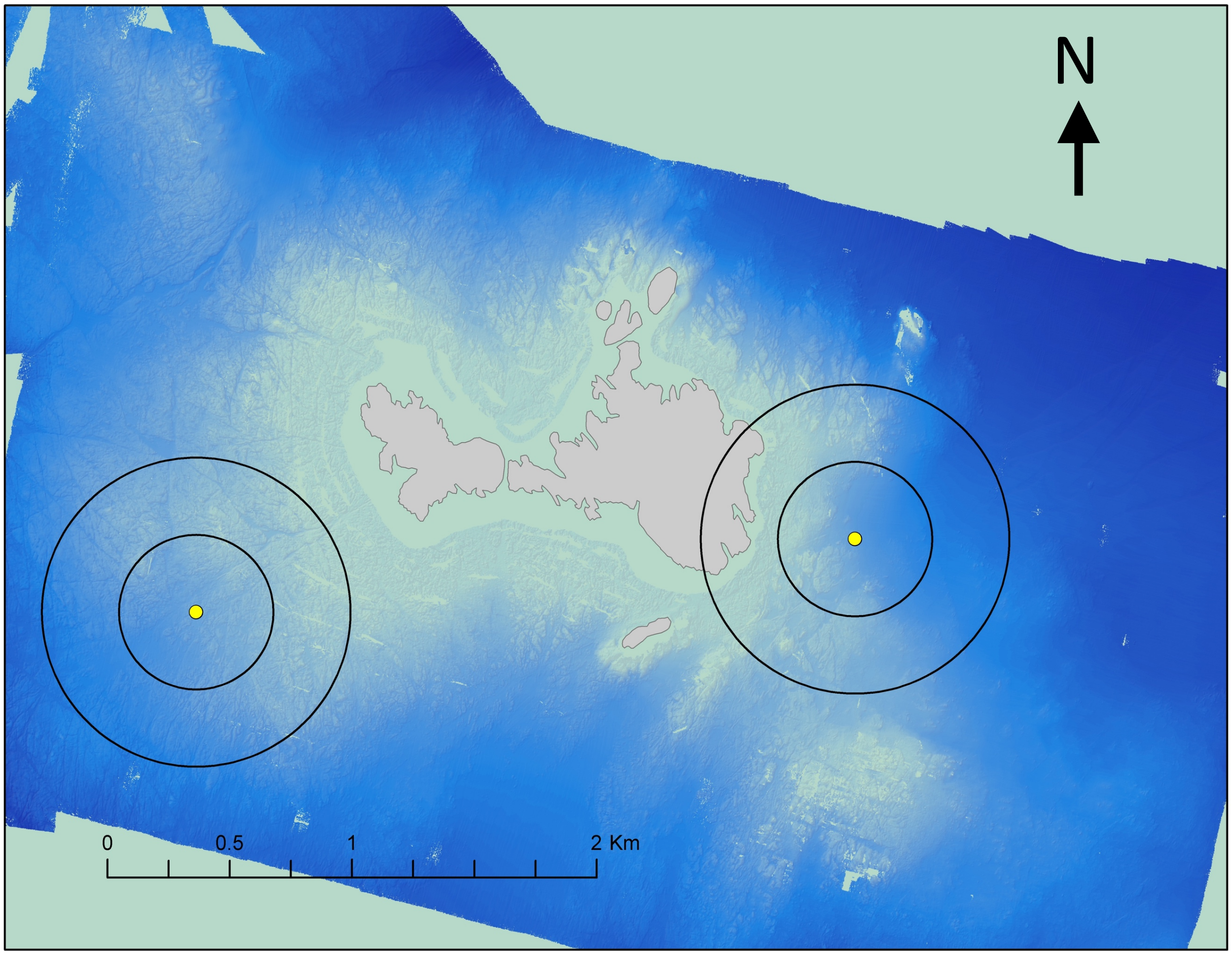

Figure S1. A map of Southeast Farallon Island (SEFI) showing the locations of the eastern and western subsurface acoustic receivers (Vemco, VR3; yellow points). Concentric circles indicate the approximate minimum (250m) and maximum (500m) acoustic tag (Vemco V16) detection range. In the 2009 event (Table 1; Fig. 3 of manuscript) Killer whales were first seen off the southern-most end of the island and gradually working their way along the western side, and eventually departing to the north-northeast. The wind directions and surface current was coming from the north. Killer whales initiated a number of killing events on pinnipeds and possibly other prey on the south, west and northwestern sides of the island. These events resulted in large areas of blood, oil and body parts dispersing downwind and sinking. At least three elephant seals and possibly other pinnipeds were killed, flung in the air and ripped into pieces. Map was created using ArcMap software (v10.3.1; ESRI; http://desktop.arcgis.com/en/arcmap/10.3/main/get-started/whats-new-in-arcgis-1031.htm)

Figure S2

A

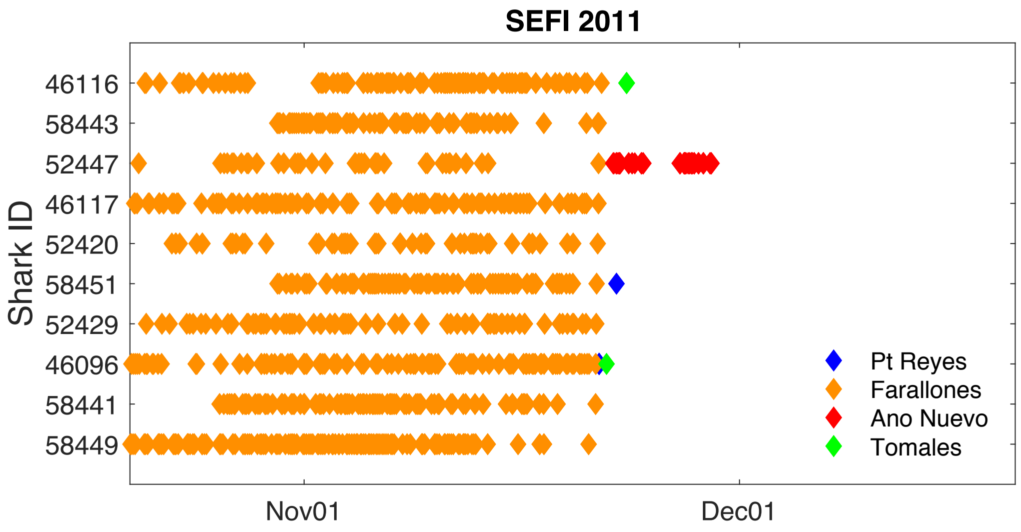

B

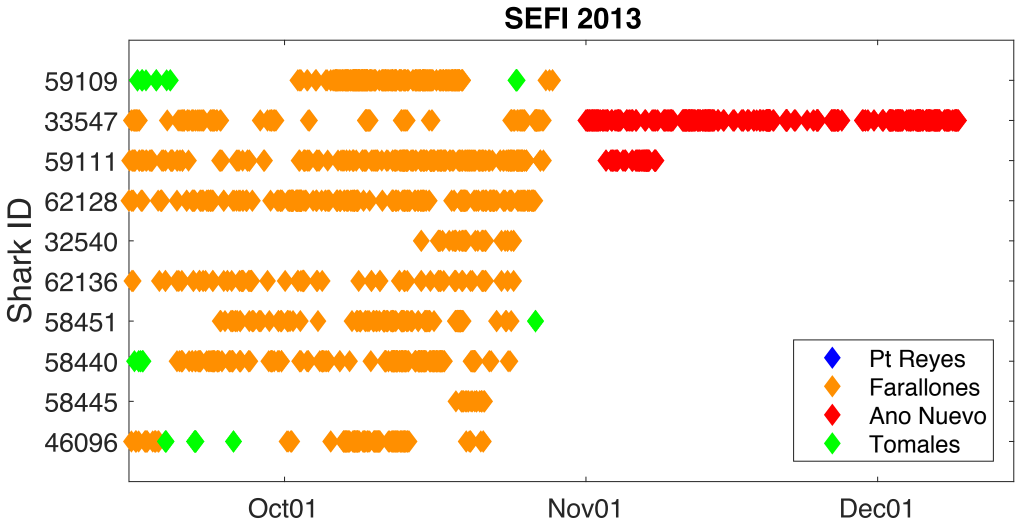

Figure S2. Flight responses of individual white sharks from Southeast Farallon Island recorded in 2011 (A) and in 2013 (B) resembled that of 2009. On October 31, 2013, 13 killer whales were observed during regular shark visual surveys from SEFI. On November 20, 2011, no surveys were conducted due to inclement weather and persistent rains, therefore there was no visual confirmation of killer whales. In both cases following these signature flight responses, no further tag detections were recorded at SEFI for the remainder of the season. Also, no further predations of pinnipeds by white sharks were observed during the remaining visual surveys in both 2011 and 2013.

Diagnostics for daily predation model

The model was fitted on a dataset of 2225 observations. Each observation was a daily visit to the observation location (Lighthouse Peak) for which we had the start and end time of the "shark watch" daily survey, and in case a shark attack was detected, we had the time, attack location within the surveyed area, prey type and the observer who recorded the event.

The model explained 18.7% of the total Deviance. We tested for overdispersion by calculating the ratio between the model deviance and the residual deviance and the value was 0.94, so approximately 1 indicating no sign of overdispersion. A similar result is obtained by fitting the model with a quasipoisson family, which results in an estimated scale parameter of 1.05. Below we report the summary statistics of the fitted model, and a multi-panel diagnostic plot showing the distributions of residuals (left panels), and scatter plots between residuals and linear predictors, and Response vs. fitted values (right panels). These plots suggest no violations of the GAM assumptions. We also report a table showing a decomposition of the variance explained by the smoothing terms suggesting that the random effect spline (s(season)) explained a significant amount of variance and was statistically different than 0. A comparison between this model and a model without the random effect spline suggest that the simpler model would have a higher AIC (+111), indicating a poorer fit.

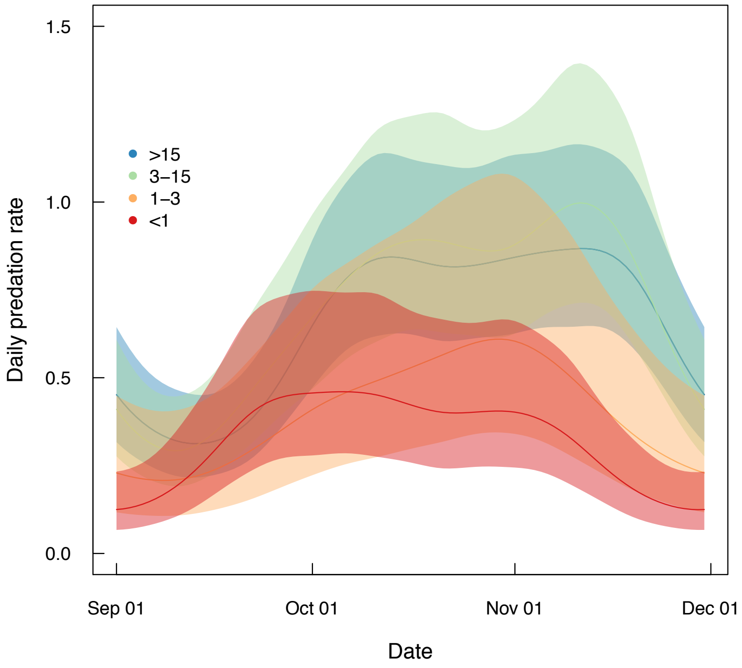

Figure S3. Seasonal *C. carcharias* kill rate as a function of the observed distance of *O. orca* activity to SEFI. The distribution of observed predations was reduced and truncated as a function of *O. orca* proximity to the common foraging ground (distance given in legend in km).

Translucent polygons indicate 95% confidence intervals of the estimated seasonal splines (solid lines).

```
Family: poisson
Link function: log

Formula:
attacks ~ s(dates, by = distanceF, bs = "cc") + distanceF + hours +
   I(hours^2) + s(season, bs = "re")

Parametric coefficients:
                Estimate Std. Error z value Pr(>|z|)
(Intercept)     -2.753704   0.317990  -8.660  < 2e-16 ***
distanceF1-3     0.234193   0.332740   0.704 0.481537
distanceF3-15    0.769599   0.253089   3.041 0.002359 **
distanceF>15     0.724944   0.243040   2.983 0.002856 **
hours            0.338876   0.068934   4.916 8.84e-07 ***
I(hours^2)      -0.017823   0.004806  -3.709 0.000208 ***
---
Signif. codes:  0 '***' 0.001 '**' 0.01 '*' 0.05 '.' 0.1 ' ' 1

Approximate significance of smooth terms:
                          edf Ref.df Chi.sq  p-value
s(dates):distanceF<1     3.880      8  22.58 3.98e-05 ***
s(dates):distanceF1-3    2.610      8  13.09  0.00101 **
s(dates):distanceF3-15   4.749      8  49.25 2.98e-10 ***
s(dates):distanceF>15    4.635      8  57.07 1.44e-11 ***
s(season)               21.450     25 123.00  < 2e-16 ***
---
Signif. codes:  0 '***' 0.001 '**' 0.01 '*' 0.05 '.' 0.1 ' ' 1

R-sq.(adj) =   0.15   Deviance explained = 18.7%
-REML = 2102.7  Scale est. = 1        n = 2225
```

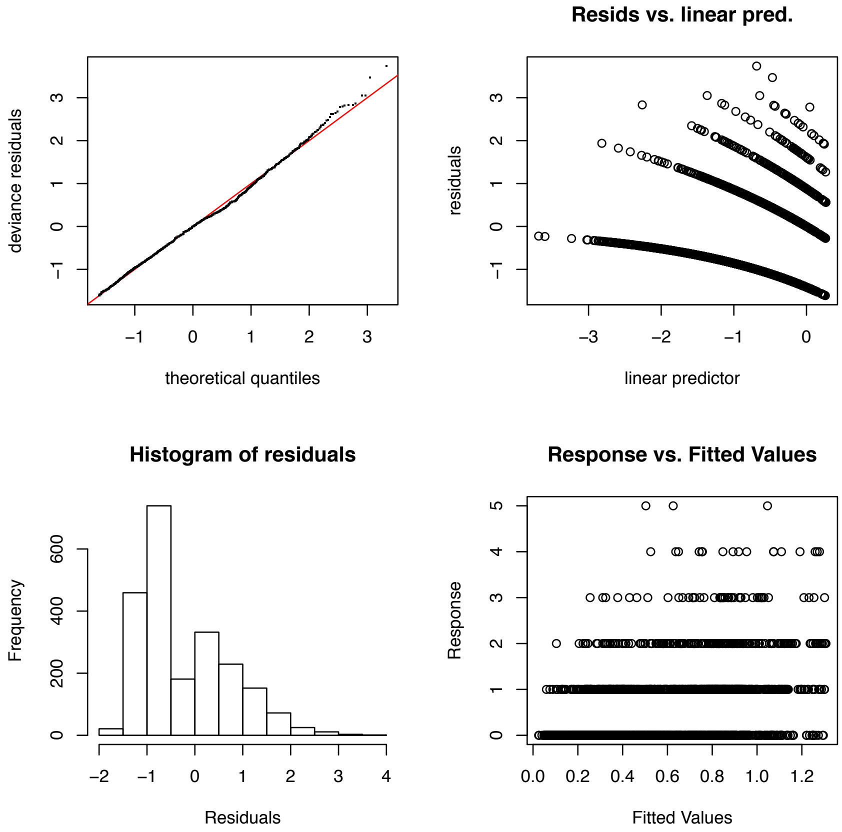

Standard deviations and 0.95 confidence intervals:

	std.dev	lower	upper
s(dates):distanceF<1	0.02143322	0.007293527	0.06298501
s(dates):distanceF1-3	0.01352695	0.004125650	0.04435142
s(dates):distanceF3-15	0.01831478	0.008112303	0.04134844
s(dates):distanceF>15	0.01565322	0.007340873	0.03337797
s(season)	0.40458327	0.286097135	0.57214004

Rank: 5/5

Linear model between log(predation rate) and log(seal count)

Call:
lm(formula = log(shark) ~ log(mir), data = pdat)

Residuals:

Min	1Q	Median	3Q	Max
-1.77350	-0.06551	0.10875	0.30541	0.59623

```
Coefficients:
                          Estimate Std. Error t value Pr(>|t|)
(Intercept)                -6.6195     1.5206  -4.353 0.000199 ***
log(mir)                    0.6350     0.2615   2.428 0.022712 *
---
Signif. codes:  0 '***' 0.001 '**' 0.01 '*' 0.05 '.' 0.1 ' ' 1

Residual standard error: 0.5459 on 25 degrees of freedom
Multiple R-squared:  0.1908,  Adjusted R-squared:  0.1585
F-statistic: 5.895 on 1 and 25 DF,  p-value: 0.02271
```

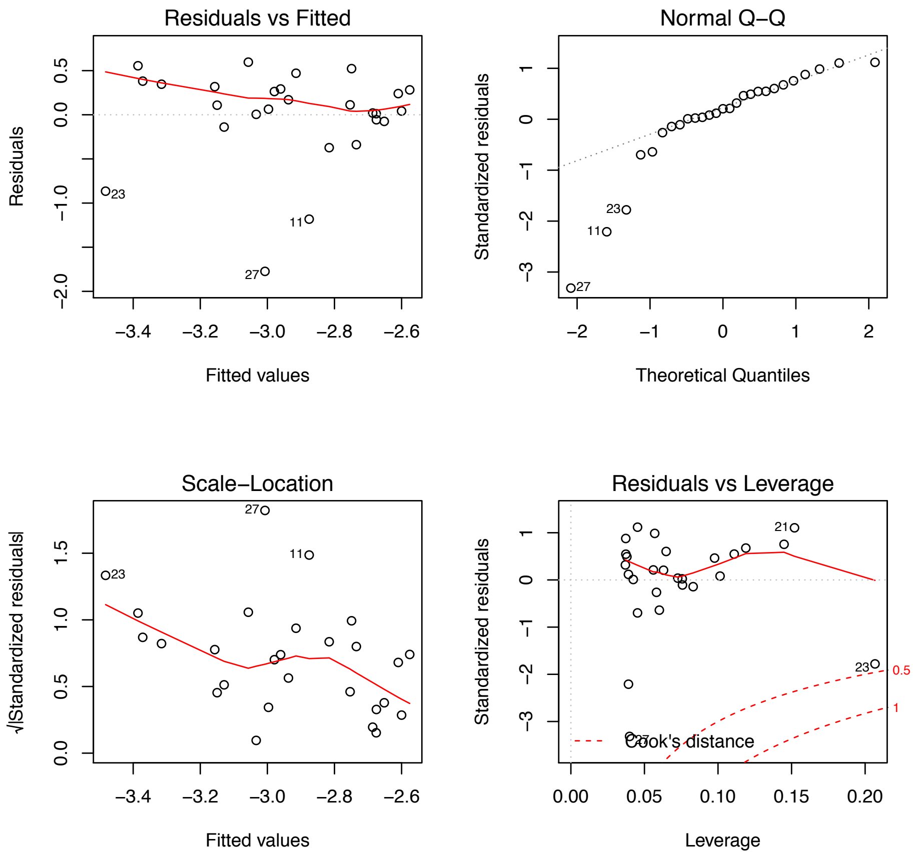

Supplement: Supplementary file 1 — Supplementary Information [file 41598_2019_39356_MOESM1_ESM.pdf]
